# Core Genome Multilocus Sequence Typing Scheme for Improved Characterization and Epidemiological Surveillance of Pathogenic Brucella

**DOI:** 10.1128/jcm.00311-22

**Published:** 2022-07-19

**Authors:** Mostafa Y. Abdel-Glil, Prasad Thomas, Christian Brandt, Falk Melzer, Anbazhagan Subbaiyan, Pallab Chaudhuri, Dag Harmsen, Keith A. Jolley, Anna Janowicz, Giuliano Garofolo, Heinrich Neubauer, Mathias W. Pletz

**Affiliations:** a Institute of Bacterial Infections and Zoonoses, Friedrich-Loeffler-Institut, Jena, Germany; b Faculty of Veterinary Medicine, Zagazig University, Zagazig, Sharkia Province, Egypt; c Institute for Infectious Diseases and Infection Control, Jena University Hospital – Friedrich Schiller University, Jena, Germany; d Division of Bacteriology and Mycology, ICAR-Indian Veterinary Research Institute, Izatnagar, Uttar Pradesh, India; e Department of Periodontology and Operative Dentistry, University Hospital Muenster, Muenster, Germany; f Department of Zoology, University of Oxford, Oxford, United Kingdom; g WOAH and National Reference Laboratory for Brucellosis, Istituto Zooprofilattico Sperimentale dell’Abruzzo e del Molise “G. Caporale”, Teramo, Italy; University of Iowa College of Medicine

**Keywords:** *Brucella*, genomic typing, cgMLST, whole-genome typing, epidemiology, core genome MLST

## Abstract

Brucellosis poses a significant burden to human and animal health worldwide. Robust and harmonized molecular epidemiological approaches and population studies that include routine disease screening are needed to efficiently track the origin and spread of Brucella strains. Core genome multilocus sequence typing (cgMLST) is a powerful genotyping system commonly used to delineate pathogen transmission routes for disease surveillance and control. Except for Brucella melitensis, cgMLST schemes for Brucella species are currently not established. Here, we describe a novel cgMLST scheme that covers multiple Brucella species. We first determined the phylogenetic breadth of the genus using 612 Brucella genomes. We selected 1,764 genes that were particularly well conserved and typeable in at least 98% of these genomes. We tested the new scheme on 600 genomes and found high agreement with the whole-genome-based single nucleotide polymorphism (SNP) analysis. Next, we applied the scheme to reanalyze the genome of Brucella strains from epidemiologically linked outbreaks. We demonstrated the applicability of the new scheme for high-resolution typing required in outbreak investigations as previously reported with whole-genome SNP methods. We also used the novel scheme to define the global population structure of the genus using 1,322 Brucella genomes. Finally, we demonstrated the possibility of tracing distribution of Brucella strains by performing cluster analysis of cgMLST profiles and found nearly identical cgMLST profiles in different countries. Our results show that sequencing depth of more than 40-fold is optimal for allele calling with this scheme. In summary, this study describes a novel Brucella-wide cgMLST scheme that is applicable in Brucella molecular epidemiology and helps in accurately tracking and thus controlling the sources of infection. The scheme is publicly accessible and should represent a valuable resource for laboratories with limited computational resources and bioinformatics expertise.

## INTRODUCTION

Brucella belongs to the *Brucellaceae* family, and members of that genus are Gram-negative facultative intracellular bacterial pathogens (1). Brucellae are highly infectious and cause brucellosis, a zoonosis reported globally (2). Typically, Brucellae are animal pathogens but can also infect humans as an incidental host, and about 500,000 new human infections occur annually (3, 4). Brucella species are listed as a category B biological warfare agent by the Centers for Disease Control and Prevention (CDC) due to their ability to undergo aerosolization (5, 6). The genus presently consists of 12 species based on the strains’ host preference and pathogenicity, including B. melitensis (mainly reported in small ruminants), B. abortus (cattle), B. suis (pigs), B. canis (dogs), B. ovis (sheep), and B. neotomae (woodrats) (7) along with six recently described novel species: B. pinnipedialis (cetaceans and seals), B. ceti (cetaceans and seals) (8), B. microti (common vole) (9), B. inopinata (breast implant) (10), B. papionis (baboons) (11), and B. vulpis (red foxes) (12).

Of all Brucella species, human brucellosis is mostly due to B. melitensis, B. abortus, and B. suis. Except for a few reports for B. canis and *B*. *ceti* (associated with marine mammals), the role of other Brucella species in human infection is questionable (13–15). Studies also showed greater variations in brucellosis prevalence in different animal species (16). In animals, cross-species transmission of Brucella spp. is also reported and can involve domestic and wild animals (17). Control of brucellosis in animal reservoirs is key to controlling infections in humans. However, the involvement of multiple bacterial species, zoonoses associated with the handling of infected animals and materials, and foodborne infections are indicative of the complex epidemiology of brucellosis. Across different continents, the prevalence of the disease is highly variable, showing an endemic nature in a few hosts in the African and Asian continents with an average prevalence range of 0 to 88.8% in sheep and goats and 0 to 68.8% in cattle (18).

Classical typing of Brucella species involves identification of different biovars. Brucella species can be classified based on serological differences, phenotypic features, and their susceptibility to phages and chemicals (7, 19). Genotypic approaches commonly employed for typing include single nucleotide polymorphism (SNP) analysis of the *rpoB* gene (20), multiple-locus variable-number tandem-repeat typing (MLVA) (21, 22), and hypervariable octameric oligonucleotide fingerprint (HOOF) variable number tandem repeats (23–25). MLVA typing is a widely used method characterizing different Brucella species (21, 26). Recently, sequence-based approaches based on multilocus sequence typing (MLST) (27, 28) that offer the possibility of web-based analysis and comparison are increasingly used for differentiating strains within the genus (29–32). Currently, two classical schemes based on nine and 21 MLST loci have been described (27, 28).

The development of typing tools that are also publicly available is of paramount importance for bacterial disease investigation, epidemiology, and outbreak surveillance. Whole-genome sequencing (WGS) has become an increasingly vital tool used by many laboratories to study the relatedness of bacterial strains in outbreaks. The most common methods include the gene-by-gene approach or SNP-based typing with or without a reference strain sequence. Typically, gene-by-gene allele calling comparison is based on the core genome (cgMLST), where only core genes are considered, or on the whole genome (wgMLST), which combines core and accessory genes for strain typing (33). For Brucella, a cgMLST scheme was initially developed based on 407 Brucella genomes (34). The scheme includes 164 targets and distinguishes Brucella down to the species and biovar level. However, the ultimate small number of target genes hampers the scheme’s utility for epidemiological analyses. At the species level, cgMLST schemes have been described only for B. melitensis. The first scheme comprised 2,704 genes (35), and the other B. melitensis-specific scheme included 2,656 genes (36). The B. melitensis schemes have proven useful for high-resolution characterization in outbreak situations (30).

This study aimed to develop a universal, web-accessible cgMLST scheme that applies to all Brucella species. We included 1,325 Brucella genomes from public repositories and used them for cgMLST scheme development and evaluation (see Table S1a in the supplemental material). Here, we demonstrated the extensibility of the scheme for defining the global population structure of the genus Brucella. We compared the scheme resolution with the core-genome SNP approach and demonstrated the scheme‘s applicability for studying brucellosis outbreaks. In addition, we have made our scheme publicly available (https://pubmlst.org/bigsdb?db=pubmlst_brucella_seqdef&page=schemeInfo&scheme_id=3) and introduced a gene-by-gene nomenclature system to harmonize the genomic characterization of Brucella strains and support the tracking of transmission chains in Brucella outbreaks. Finally, we benchmarked a data set of genomes with various read depths and examined the impact of read depth on the typing results of cgMLST.

## MATERIALS AND METHODS

### Brucella genome retrieval and processing.

In this study, we included 1,325 Brucella genomes from public repositories and used them for cgMLST scheme development and evaluation (see Table S1a in the supplemental material). For scheme development, we downloaded the Brucella genomes from the National Center for Biotechnology Information (NCBI) RefSeq database following the taxonomy number 234. Of the 783 genomes downloaded (March 2021), we excluded 57 genomes that were based only on sequencing technologies prone to high error rates, e.g., 454/Roche, ONT, and Ion Torrent. We also excluded 92 genomes with completeness scores less than 95% and contamination and heterogeneity scores more than 5% using CheckM v1.1.3 (37). The remaining genomes (*n* = 634) were used for taxonomical analysis by estimating the level of nucleotide similarity with FastANI v1.3 (38), and by performing a protein-based phylogeny using up to 400 universal bacterial proteins with PhyloPhlan v0.43 (39). We then used Mash v2.1 (40) for species confirmation. As a result, we included only 612 genomes for scheme development (Table S1a).

For scheme evaluation, we additionally used 600 random genomes from the Short Read Archive (SRA) database, for which the metadata on isolation sources were available and the sequencing was done with Illumina platforms (Table S1a). The fasterq-dump module of SRAtoolkit v2.9.2 was used to obtain files in FASTQ format (https://github.com/ncbi/sra-tools), fastp v0.20 was used to check the quality of the sequencing reads (41), and shovill v1.0.4 (SPAdes v3.12 [42]; flags –trim –minlen 500 –mincov 5) was used to perform genome assembly (43). The quality of genome assemblies was checked with Quast v4.3 (44), and we retained genomes with an *N*_50_ greater than 20,000 bases, and a number of contigs less than 1,000. Finally, Mash v2.1 was used to confirm the species of all genomes.

To evaluate the scheme for outbreak analysis, we reinvestigated published outbreak strains including 37 B. melitensis strains involved in outbreaks in central Italy (35), and a further 76 B. abortus strains implicated in several outbreaks in Northern Ireland (45) (Table S1a). The raw reads were obtained from the SRA database and processed using fastp v0.20, shovill v1.0.4, Quast v4.3, and Mash v2.1 as mentioned above.

### Development of cgMLST for Brucella.

We first performed high-resolution phylogeny of the genus. We identified consensus SNP positions with NASP pipeline v1.1.2 (46), for which we used as input genome assemblies aligned with NUCmer (47) to the reference genome of strain 16M. SNP positions falling into the duplicated regions were masked. Consensus SNPs were identified in each genome relative to the reference genome. We then used RAxML v8.2.9 (48) to perform a maximum-likelihood phylogenetic analysis using ascertainment bias correction and the gamma model of evolution.

Next, we used the genome of strain 16M (GenBank accession numbers NC_003317.1 and NC_003318.1; 25 October 2020) as a reference. The cgMLST Target Definer tool v1.5 of SeqSphere+ v7 was then applied in default mode to remove short, multicopy, and overlapping genes as well as genes with internal stop codons from the reference genome (49). As a result, 2,763 genes were kept. We then used these genes to genotype the quality-controlled 612 genomes from the RefSeq database. The percentage of the good targets was then estimated per each genotyped genome. A threshold of 98% was applied. Genes with an allele call rate below 98% were manually excluded from the final cgMLST scheme. The default settings of SeqSphere+ v7 for allele calling were used, including BLAST detection with BLASTN v.2.2.12 at sequence identity above 90% and sequence overlap above 99%.

### Application of Brucella cgMLST.

SeqSphere+ v7 (49) was used to detect cgMLST genes and allele calls. Gene detection was done using BLASTN (50) at a sequence identity of >90% and an overlap of >99%. Allele assignment involved only intact genes without frameshifts or sequence ambiguities. In-frame insertions or deletions of up to three codons relative to the reference genes were allowed. The cgMLST allelic profiles were then created using a combination of alleles identified for each gene. Finally, the allelic profiles were compared (uncalled genes were ignored in pairwise comparisons), and the resultant pairwise distances were used to generate a neighbor-joining (NJ) tree and a minimum spanning tree (MST) using SeqSphere+. The Simpson index of diversity and adjusted Wallace test of congruence were calculated using the Comparing Partitions tool (51). For the outbreak data set, we calculated core genome SNPs using the snippy pipeline v 4.6.0 (https://github.com/tseemann/snippy) with default settings.

### Allele calling of cgMLST at various sequencing depths.

We used a subset of 40 genomes available in the SRA database to examine the impact of sequencing depth on the allele calling rate of cgMLST and cluster definitions. Briefly, we assessed genome quality using fastp v0.20.0 in standard mode (41) and SeqKit v2.2.0 (52) to generate statistics of FASTQ files including Phred quality scores and total bases sequenced. ConFindr v0.7.4 (53) was then used to detect contamination. For ConFindr, the rMLST database (54) was also employed to identify contaminants based on variations in ribosomal proteins.

Next, we created a data set *in silico* with read depths between 10- and 100-fold. For this, we used rasusa v0.6.1 (55) to randomly downsample sequencing reads to the required coverage using the genome size of the B. melitensis strain as a reference (flag -g 3.2MB). BBMap (version dated 24 February 2022) (56) was then used to independently estimate the obtained sequencing depth and breadth of coverage.

For each downsampled data set, SKESA v2.4.0 (57), MEGAHIT v1.2.9 (58), and SPAdes v3.15.4 (42) were used for assembly. All assemblers were used with default settings except SPAdes, which was run with the flag (--careful) to reduce the number of mismatches and short indels. No refinement steps such as read trimming, polishing, or scaffolding were added before or after assembly. The 1,200 genomes generated by the three assemblers were allele called based on the BLASTN algorithm (50) using the SeqSphere+ program (49) and default settings (90% identity and 99% alignment to reference sequences required).

### Data availability.

Genome sequence data examined in this study are publicly available under the accession numbers given in Table S1a.

## RESULTS

### Definition of a cgMLST scheme for Brucella*-*wide genotyping.

**FIG 1:**
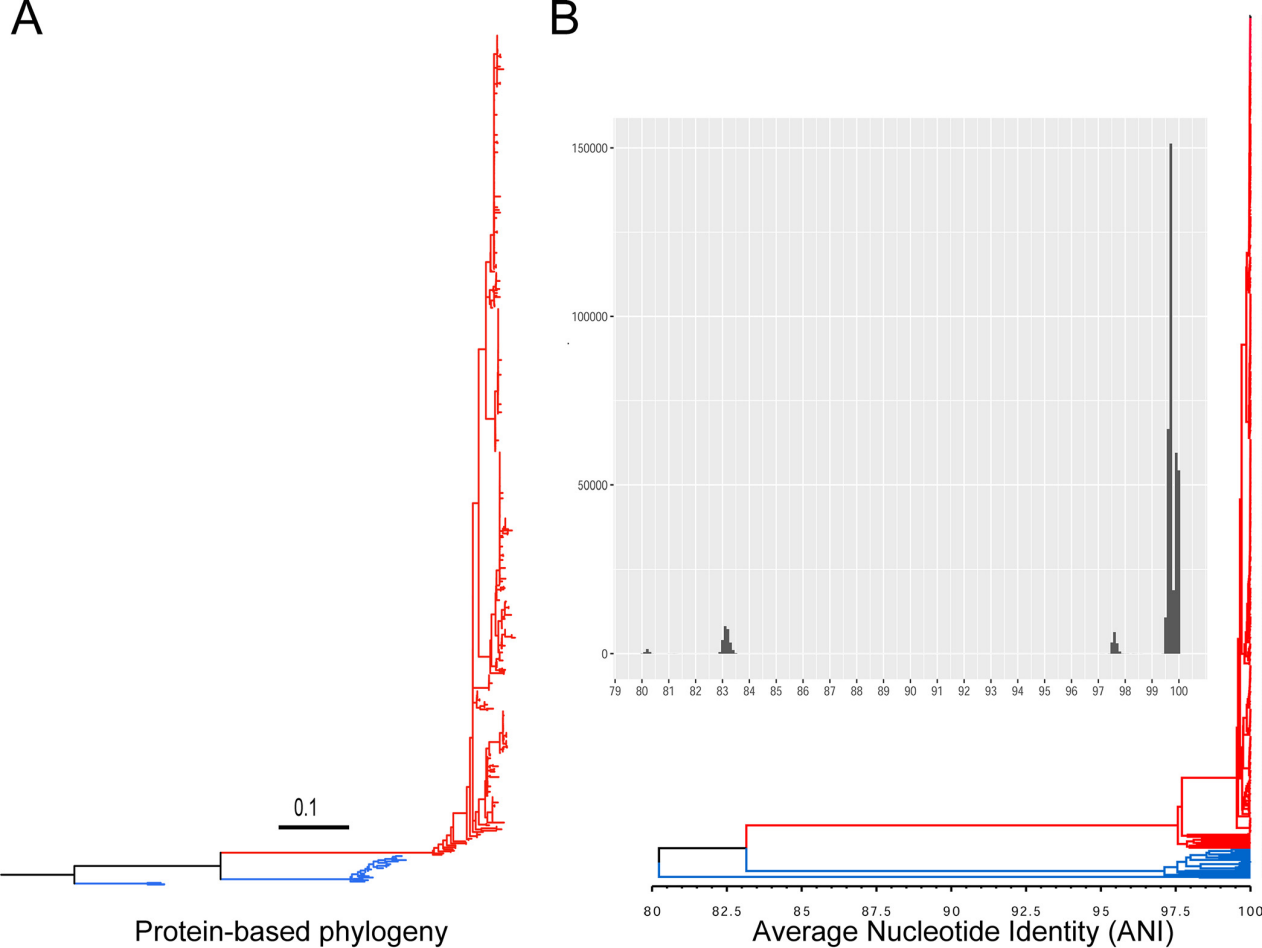
Taxonomic classification of 634 Brucella genomes downloaded from the NCBI Reference Sequence (RefSeq) database (March 2021). (A) A midpoint-rooted maximum-likelihood (ML) phylogeny of Brucella genomes calculated with PhyloPhlAn. The phylogeny is based on the concatenated alignment of 24,110 amino acid positions of up to 400 universally conserved bacterial proteins. (B) The results of pairwise average nucleotide identity (ANI) between all Brucella genomes calculated with FastANI and plotted with bactaxR. The distribution of the ANI values is represented in the histogram, and the relatedness between all genomes is illustrated by a dendrogram created using the average linkage hierarchical clustering method where the tree height corresponds to ANI similarity. The red branch denotes the genomes of all Brucella species while the blue branches denote divergent genomes from the species *B. intermedia* (*O. intermedium*) and Brucella pituitosa, as explained in the text.

To begin with the scheme setup, we first quality-filtered 634 Brucella genomes from the Reference Sequences (RefSeq; accessed March 2021) database and used them to define the overall genetic diversity of the genus (see Materials and Methods and see Table S1a in the supplemental material). The midpoint-rooted maximum-likelihood (ML) phylogeny, supported by the average nucleotide identity (ANI) analysis, divided the RefSeq genomes into two distinct groups: a small divergent group (group I) of 22 genomes from the species Brucella intermedia (formerly Ochrobactrum intermedium [59]) and Brucella pituitosa and a large group (group II) with 612 genomes (Table S1a and b). The genomes of the second group include the following Brucella species: B. melitensis, B. abortus, B. suis, B. canis, B. ovis, B. neotomae, *B. ceti*, *B. pinnipedialis*, B. microti, and *B. inopinata* (Table S1a). These species fall collectively into a single genomic species as described previously (60), with ANI values greater than 97%. Of the 612 genomes, 601 (98.2%) had ANI concordance more than 99% compared to the B. melitensis reference 16M genome (Fig. 1). ANI results between groups I and II were 80 to 83% (Fig. 1). Based on this divergence level, we excluded the 22 genomes of group I and focused our genome typing scheme on group II Brucella (Fig. 1).

We used 612 Brucella genomes (group II) to perform a high-resolution phylogeny based on the genomes’ nucleotide content (Fig. S1). We determined 159,572 SNP positions (4.84%) in the genomes with the NASP pipeline (46). The phylogenetic analysis, consistent with previous reports (2, 26, 28, 61), showed characteristics of the global Brucella phylogeny, including (i) simultaneous evolutionary radiations of several descendant lineages, each lineage including a single or pair of species; (ii) clear separation of the genomes of each species into a distinct clade; (iii) ancestral relationships between some Brucella species, such as B. canis, was shown to have evolved later from the species B. suis, as was B. pinnipedialis in relation to *B. ceti* (2, 62); and (iv) a genetically more distant group of genomes (*n* = 11 genomes) positioned at the base of the phylogenetic tree, including one genome of the species *B. inopinata* and another 10 genomes of unassigned species. We note that 52 genomes in the data set were not originally reported for a specific Brucella species and that potential mislabeling of the species was identified (Table S1a). These results indicate that the 612 genomes cover the known global population structure of Brucella and are therefore suitable to set up a cgMLST scheme. However, two Brucella species were not considered: *B. papionis*, for which no assembly is available, and *B. vulpis*, for which the only two available genomes were not incorporated in the RefSeq database because of anomalous assembly (March 2021). The compiled genomic data set represented diverse geographic origins (54 countries of the six continents), isolation from diverse ecological sources including humans (*n* = 184) and animals (*n* = 291), and diverse isolation times (from 1931 to 2020; Table S1a).

Next, we used the 612 genomes to create a “soft defined” cgMLST scheme as described previously (63). Briefly, SeqSphere v7 removed 340 genes (192 incomplete, 16 repetitive, and 132 overlapping genes) from the reference genome (strain 16M; accession numbers, NC_003317.1 and NC_003318.1; 25 October 2020; total genes, 3,103). The retained 2,763 genes were then used to genotype the 612 genomes. The genes that were present and allele typed in at least 98% of the 612 genomes were placed in a core genome scheme, whereas the remaining genes were moved to an accessory genome scheme. As a result, 1,764 genes formed the core genome MLST scheme. The genes range in size from 60 to 4,134 bp, with a total length of 1,556,385 bp, covering 47.2% of the reference genome size and 56.8% of the reference genes with coding DNA sequences (Table S1c). The accessory genome MLST scheme included 1,131 genes with a total length of 1,093,278 bp.

### Evaluation of cgMLST for high-resolution genotyping of Brucella genomes.

To evaluate the performance of the novel scheme, we used an additional 600 genomes randomly obtained from the Short Read Archive (SRA) database, representing strains collected between 1940 and 2020: 184 were from humans, 450 from animals, and 42 from unknown hosts. The strains originated from 49 countries on the six continents. The 1,764 cgMLST targets were highly conserved in the 600 genomes, with an average allele call rate of 99.5% (median, 99.9%; standard deviation, 0.7%). The average number of variants found for each cgMLST gene was 12 ± 6.4 variants (range, 1 to 47), with a total number of SNP positions ranging from 0 to 177 per gene and a total of 49,705 SNP positions in all genes (Table S1c). The length of cgMLST genes correlated directly with the number of variants detected for each gene (Table S1c).

The cgMLST resulted in high-resolution genotyping. The 600 genomes were clustered into 476 cgMLST sequence types (cgST), ignoring missing values for pairwise comparisons. This resulted in a Simpson index of discrimination of 0.998 (95% confidence interval [CI] = 0.997 to 0.999). Analysis of the whole-genome SNP provided a slightly higher resolution than that of the cgMLST. We detected 92,651 SNPs across the 600 genomes. The number of unique SNP profiles was 531 (Simpson‘s index = 0.999, 95% CI = 0.999 to 1.000). Considering only SNP sites in the 1,764 cgMLST targets led to identifying an almost similar number of SNP profiles as with core genome MLST (*n* = 482).

Finally, the cgMLST had a higher resolution than the classical MLST schemes of the genus Brucella. In total, the 600 genomes were classified into 40 ST using the 9-gene MLST scheme, whereas 596 of the 600 genomes (four genomes of undetermined MLST21 ST) were classified into 65 ST using the 21-gene MLST scheme, resulting in a Simpson index of diversity of 0.868 (95% CI = 0.853 to 0.882) and 0.898 (95% CI = 0.883 to 0.914), respectively (Table S1a).

### Reanalysis of Brucella outbreaks with the novel scheme.

We used the developed scheme to characterize published sets of epidemiologically linked cases of brucellosis involving B. melitensis and B. abortus. For B. melitensis, we included the genome of 37 strains of known epidemiological links described in a previous study (35). The strains were collected from a single outbreak in 21 farms from four provinces in central Italy (35). The study compared the resolution of core genome SNPs and classical MLVA typing with a B. melitensis-specific cgMLST scheme. The results showed that two distinct clusters were involved in the outbreak and that the inter- and intracluster distance resolution achieved with WGS methods was higher than that with classical MLVA.

We retrieved the raw sequence data from this previous study and reprocessed them (see Materials and Methods). Because the assembly workflow in our study differed from that of the original study, we first wanted to examine the effects of assembly on the cgMLST results. Therefore, we first reapplied the previously described B. melitensis-specific cgMLST (https://www.cgmlst.org/ncs/) to the genomes (35). While the results were highly reproducible, there were some minor differences in the number of different alleles between the genomes in each cluster (Fig. S2). The allelic distances within the two clusters were higher than previous results (missing values ignored for pairwise comparisons; Fig. S2). Cluster 1 had a mean difference of 3.8 (range 0 to 15) while cluster 2 had mean allelic differences of 5 alleles (range 2 to 7) (Fig. S2). One hundred sixty-one gene variants represent the intercluster genetic distance.

**FIG 2:**
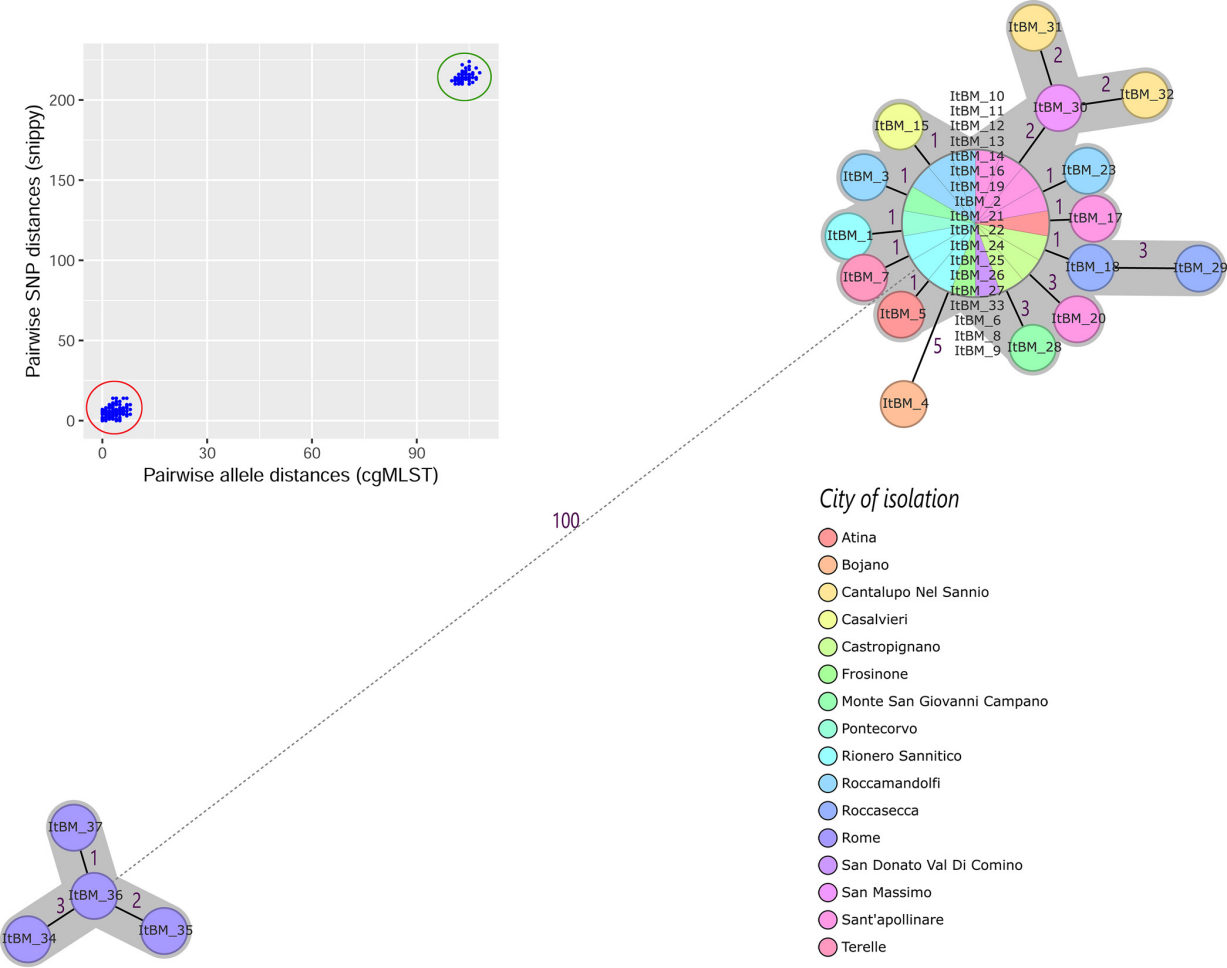
Minimum spanning tree (MST) calculated for 37 Brucella melitensis genomes with known epidemiological linkage using 1,764 Brucella-wide core genome MLST targets. The MST was generated with Ridom SeqSphere, ignoring missing values in pairwise comparisons. Each circle represents a unique cgMLST profile and is labeled according to the strain’s city of isolation. The circle size is proportional to the number of genomes per each cgMLST genotype. The number of different alleles between cgMLST profiles is indicated on the connecting lines. Solid and dashed lines represent allele differences below and above 10, respectively. Clusters are highlighted with gray-shaded areas based on a threshold of three allele mismatches between any two neighbors. The inset box shows the comparison of cgMLST allele distance and core genome SNPs for each genome pair. Genomic distances (cgMLST alleles and SNPs) within and between outbreak strains are highlighted by red and blue circles, respectively. For a phylogeny based on the core genome SNPs, we refer the reader to the original publication (35).

Next, we applied the Brucella-wide cgMLST to the 37 genomes. The results show that at least 99.8% of the scheme targets were detected in each genome, with all targets detected in 29 genomes (100% allele call rate) (Table S1a). The typing results were congruent with the original study, defining the two genetic clusters involved in the outbreak using a clustering threshold of three different genes between any two neighboring isolates; one isolate had 5 genes different from the closest neighbor (Fig. 2). The 37 genomes were indexed into 20 cgST (missing values were ignored for pairwise comparisons). Eighteen of the 37 genomes formed a single central cgST of cluster 1. The mean genetic variation of cluster 1 was 1.9 ± 1.9 alleles (median, 1; range, 0 to 8) and of cluster 2 was 2.3 ± 0.8 alleles (median, 2.5; range, 1 to 3) (Table S1d). The intercluster allelic variation was 100 distinct alleles. These results agree with the typing output of the B. melitensis-specific scheme, indicating that the two schemes produce comparable resolutions suitable for identifying the closely related strains.

**FIG 3:**
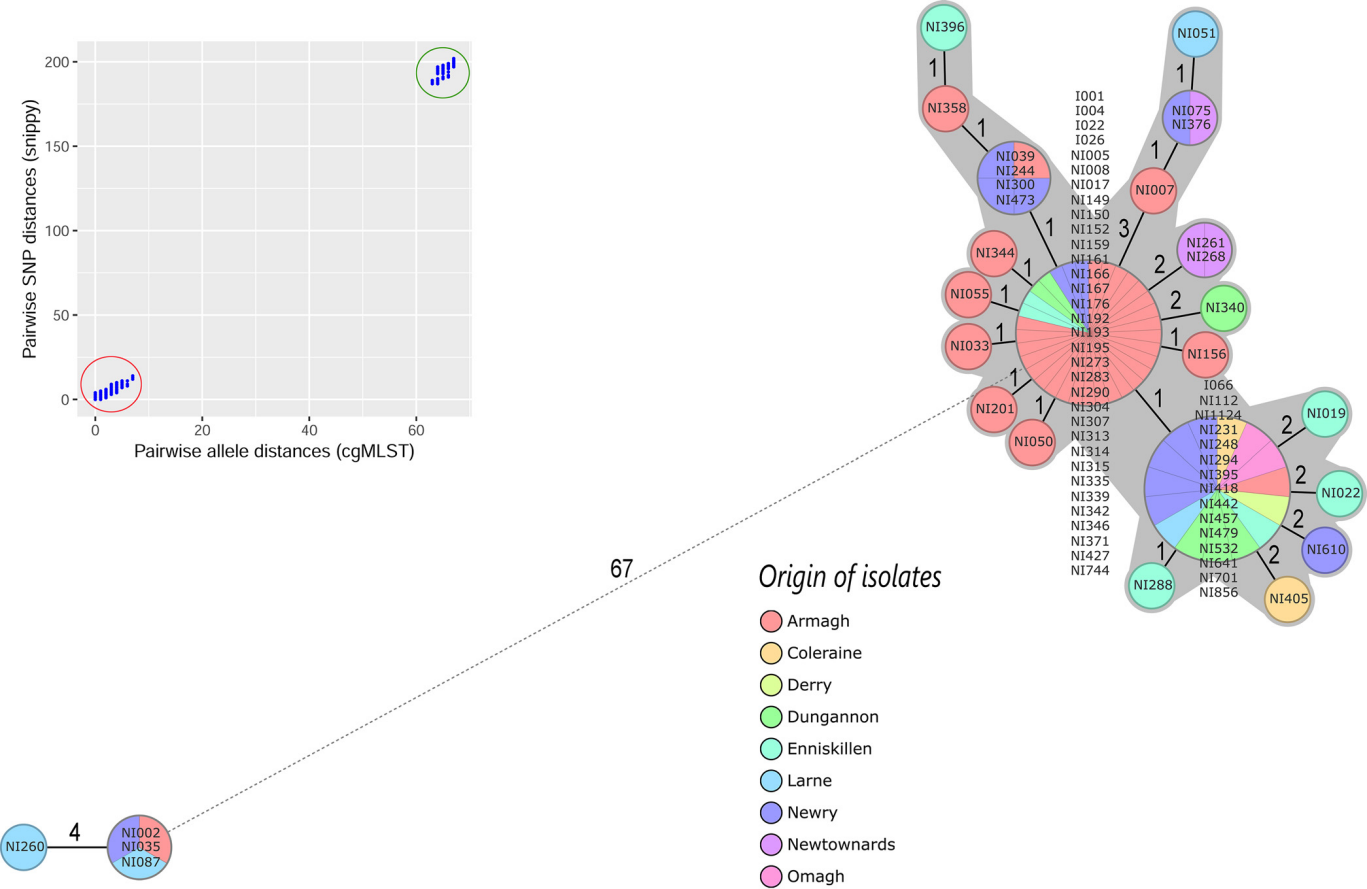
Minimum spanning tree (MST) calculated for 76 Brucella abortus genomes using 1,764 Brucella-wide core genome MLST targets. The MST was generated with Ridom SeqSphere, ignoring missing values in pairwise comparisons. Each circle represents a unique cgMLST profile and is labeled according to the origin of strains. The circle size is proportional to the number of genomes per each cgMLST genotype. The number of different alleles between cgMLST profiles is indicated on the connecting lines. Solid and dashed lines represent allele differences below and above 10, respectively. Clusters are highlighted with gray-shaded areas based on a threshold of three allele mismatches between any two neighbors. The inset box shows the comparison of cgMLST allele distance and core genome SNPs for each genome pair. Genomic distances (cgMLST alleles and SNPs) within and between outbreak strains are highlighted by red and blue circles, respectively. For a phylogeny based on the core genome SNPs, we refer the reader to the original publication (45).

For B. abortus, we reinvestigated the genomes of 76 strains involved in brucellosis outbreaks in cattle in nine different locations related to Divisional Veterinary Offices (DVOs) in Northern Ireland from 1991 to 2011 (45). The strains had limited genetic distances and were previously characterized with classical MLVA and WGS-based SNP analysis (45, 64). Two phylogenetic lineages were characterized, a small lineage of 4 strains and a large lineage of 72 strains. The cgMLST classified the genomes into 23 cgST where the allele call rate was 99.8% to 100% in all genomes (only 1 to 3 targets were missing in 7 genomes). In congruence with the SNP analysis, the MST of the cgMLST profiles indicated a limited heterogeneity for all genomes (Fig. 3). In addition, the two genetic lineages were identified with 67 to 72 differing alleles between the two lineages. Lineage 1 included two cgST that were distant by four alleles. Lineage 2 included 72 genomes with mean allele differences of 1.6 ± 1.5 (median, 1; range, 0 to 8 alleles) and a maximum of three different genes between any two neighboring isolates (Fig. 3 and Table S1e). These cgMLST results confirm the results of the previous whole-genome SNP analysis, indicating comparable performance (45).

### Deciphering the population structure of Brucella with cgMLST.

**FIG 4:**
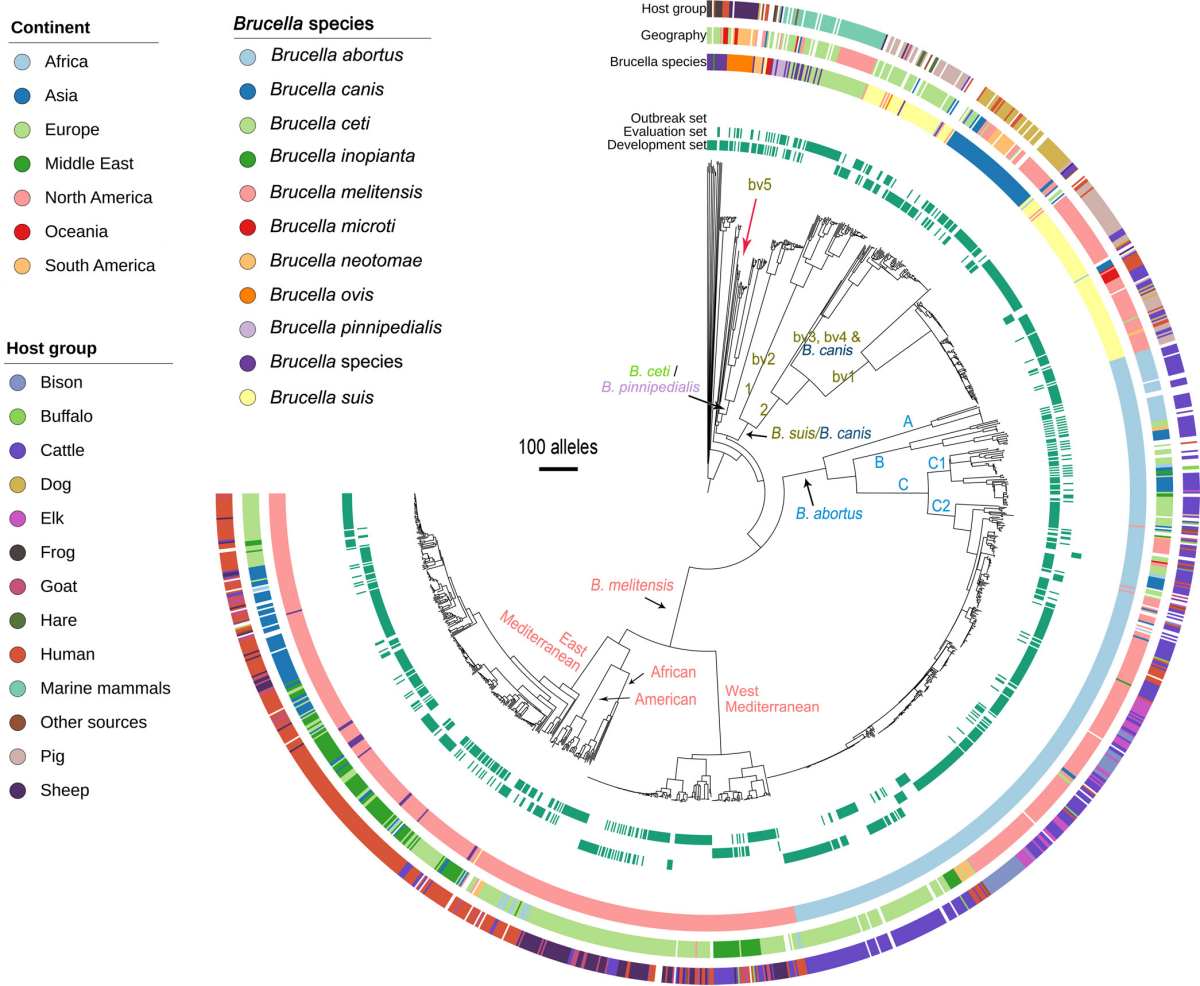
Neighbor-joining tree constructed for the 1,322 Brucella genomes based on the cgMLST allelic profiles, deciphering the characteristic population structure of pathogenic Brucella. Tree visualization was performed using iTOL.

We deciphered the population structure of the genus Brucella using cgMLST (Fig. 4). Therefore, we used 1,322 (out of 1,325) genomes with an allele calling ratio over 90%. The characteristic population structure of Brucella as determined by cgMLST was consistent with the core genome SNP analysis (Fig. S3), demonstrating that each species is clearly divided into a distinct clade and that the individual lineages of each species are well resolved. In B. melitensis (*n* = 498 genomes), four main lineages were identified: (i) the West Mediterranean lineage, which was detected in the Middle East and Europe; (ii) the African lineage in Africa; (iii) the American lineage in North and South America and Europe; and (iv) the East Mediterranean lineage in Asia, Europe, and the Middle East. Here, the Mediterranean lineages were more abundant in the B. melitensis data set (90.7%, *n* = 452 genomes). In B. abortus (*n* = 472 genomes), the three main clades, A, B, and C, were identified. Clades A and B were less common and restricted to African countries (9.7%, *n* = 44 genomes). These two clades represent early-branching groups, in contrast to clade C, which represents a relatively recent branch of B. abortus. Clade C (subdivided into subclades C1 and C2) has a wide geographic distribution (90.6%, *n* = 428 genomes). In B. suis, two clades were identified: clade 1, including biovar 2 genomes, was mainly attributed to Europe, while clade 2, comprising biovars 1, 3, and 4 as well as B. canis genomes, was mostly attributed to North and South America, Asia, and Tonga. In addition, two B. suis genomes corresponding to MLST21 ST19 (B. suis biovar 5) formed a separate branch from the other B. suis genomes. The close phylogenetic relationship of B. suis and B. canis was also demonstrated with cgMLST, as was the close relationship between *B. ceti* and *B. pinnipedialis*, which were isolated mainly from marine mammals. B. ovis comprised five genomes from MLST21 ST13 that form a distinct lineage. Overall, these results show that the new scheme provides sufficient resolution to delineate the global population structure of Brucella strains.

Among the 1,322 genomes examined with the scheme, we observed nearly identical cgMLST profiles detected in different countries/laboratories (Table S1f). This included 58 genomes classified into 12 cgMLST cluster groups with fewer than three different alleles. Clusters included reference strains of the species and vaccine strains processed remotely in different laboratories. Unfortunately, the detailed epidemiological information on all genomes was sparse. However, some were reported in previous studies (31, 65), tracing strains back to patients with travel or migration history. This was shown, for example, in patients from Norway and Sweden who had a travel history to countries of endemicity such as African or Middle Eastern countries, but patients with no travel history also were reported, possibly indicating hidden transmission routes, e.g., via food vehicle or illegal animal trade. Similar cgMLST profiles were also observed in different host species, confirming transmission of Brucella between hosts. Taken together, these results indicate that the typing efficacy enables correlation with the source country of infections and thus that trace-back analysis is possible based on related strains reported after a time span in different regions, laboratories, or hosts.

### Impact of sequencing read depth on allele calling rate and cluster analysis of cgMLST.

We aimed to evaluate the impact of sequencing depth on allele calling rate and cgST cluster definition. To this end, we collected 40 genomes from the SRA database that were sequenced with paired-end libraries with read lengths of 100, 150, 250, and 300 bp. This was based on our observation that 92.5% (*n* = 24,18) of Brucella genomes in the SRA database (accessed 31 May 2022, WGS data = 2,965 genomes) were based on Illumina platforms and were predominantly produced using paired-end libraries (94%, *n* = 2,278). As such, we based our evaluation procedure on Illumina data. For all included genomes, no evidence of contamination was found using the ConFindr tool (53) and the rMLST database (54). In addition, at least 80% of raw reads had Phred quality scores above 30. The estimated sequencing depth for all genomes was higher than 100-fold.

Based on these 40 genomes, we benchmarked a data set of downsampled reads. We randomly downsampled the genomes to obtain target read depth in the range of 10- to 100-fold (Table S1g). The outcome of this random downsampling procedure was independently evaluated with the BBMap mapping tool, and we found high agreement between the target depth (i.e., the depth specified by downsampling) and the depth reported by BBMap (56) (*R*^2^ > 99.9%; Pearson test, *P* value < 2.2e−16), with an absolute mean difference of 0.74 ± 1.05 (Table S1g). In all genomes, the breadth of coverage was higher than 99.2% over the two Brucella chromosomes (Table S1g). Using this benchmarked data set, we performed *de novo* assembly using three assembly software programs, SPAdes (42) (correction mode), SKESA (57) (default settings), and MEGAHIT (58) (default settings). The final data set comprised 1,200 genomes (40 genomes × 10 various read depths × 3 assembly software programs). The results show that the contiguity statistics of all assemblers did not show significant improvement at read sequence depth of more than 40-fold (Table S1h). Above this depth, the highest *N*_50_ was reported with SPAdes (mean, 224,714 bp) compared to MEGAHIT (mean, 158,700 bp) and SKESA (mean, 155,671 bp) (Table S1h). At low read depth (10- and 20-fold), SPAdes and MEGAHIT generally showed higher contiguity values (Table S1h). At read depth above 30, the three assemblers produced genomes with overall contig size covering >98% of the reference genome. Despite the good contiguity metrics of all assemblers, three genomes (ERR471311, ERR471313 [*B. ceti*], and SRR11449060 [B. melitensis]) produced by SPAdes and MEGAHIT were found to deviate from the reference genome size by more than 20% (i.e., the total genome size was 4.1 to 7 Mbp compared to 3.2 Mbp of the reference genome), and GC content deviated from the reference genome by more than 10% (i.e., GC content was 44 to 50% compared to 57.2% of the reference genome) (Table S1h).

**FIG 5:**
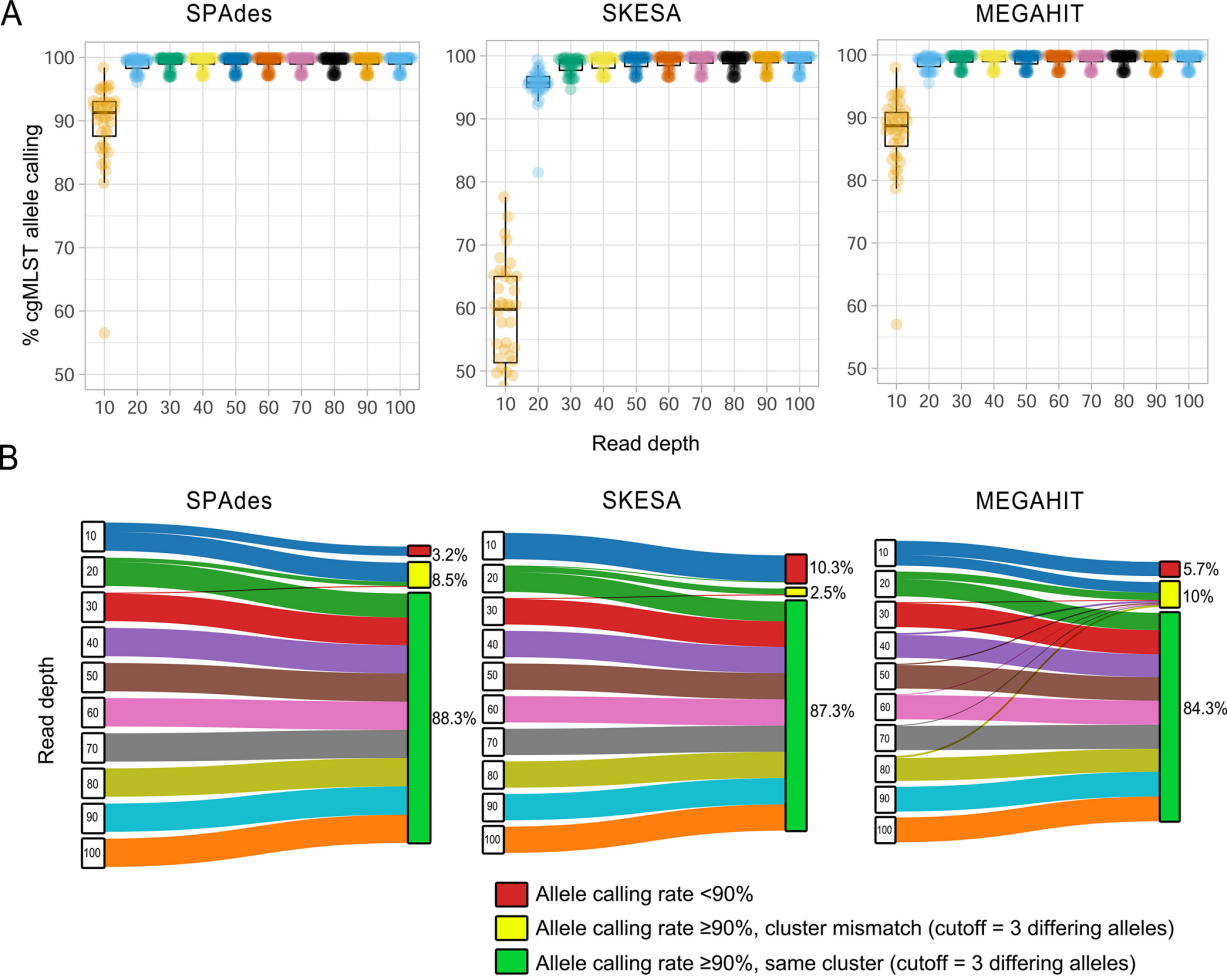
Effect of various sequencing depths and assembly software on allele calling rate and cluster analysis with Brucella cgMLST. (A) Box plots of the mean percentage of allele calling rate according to assembler for data generated with different coverage depths (*n* = 40 genomes per group). (B) Alluvial plots showing the frequency of the effect of read depth on clustering results of cgMLST data.

For the 1,200 genomes generated by the three assemblers, we determined the number of cgMLST loci found and allele called. We clustered the genomes with an allele calling rate above 90% into cluster types at fewer than three differing alleles. Cluster analysis was consistent for SPAdes and SKESA genomes at a coverage depth of 40 or more, i.e., the downsampled genomes from the same sample were grouped into the respective cluster type (CT) (Fig. 5). However, for MEGAHIT, a higher sequencing depth of 90-fold was required to avoid inconsistencies in cluster definition of three (out of 40) genomes (Fig. 5). Above these depth thresholds, cluster definition (3 differing alleles) was highly reproducible with complete agreement between the three assemblers (adjusted Rand coefficients and adjusted Wallace from two comparison directions = 1) (Fig. 5). However, reproducible assignment of the cgST profiles (zero differing alleles) across different assemblers showed incomplete agreement even at higher depth values. SKESA showed the best overall reproducibility (reproducible cgST for 38 out of the 40 genomes at read depth of >40-fold), followed by SPAdes (25 out of 40 genomes; read depth, >40) and MEGAHIT (25 out of 40 genomes; read depth, >40) (Table S1h). Additionally, the cluster mismatch was minimal in SKESA assemblies compared to SPAdes and MEGAHIT (Fig. 5). On the other hand, SKESA genomes had the highest number of unidentified alleles at low read depths (10- and 20-fold) due to the high fragmentation of the genome (Fig. 5).

### Scheme incorporation in PubMLST Brucella database.

The cgMLST scheme was made accessible via the PubMLST Brucella species database (https://pubmlst.org/organisms/brucella-spp) (66). An allele nomenclature database for the proposed scheme was also integrated. The scheme is also accessible via the PubMLST RESTful application programming interface (https://rest.pubmlst.org) (67). Classification of the cgMLST allelic profiles is performed using the single-linkage method with cutoff thresholds defined hierarchically at 200, 100, 50, 25, 10, 5, and 3 different alleles in order to reveal different levels of relationships between the strains.

## DISCUSSION

Brucellosis is a highly contagious bacterial zoonosis that affects numerous animal species and can cause significant economic losses in livestock, as well as severe illness and death in humans (16, 68). The classical (core) species of the genus Brucella are genetically monomorphic with limited recombination and horizontal gene transfers, which implies the importance of whole-genome sequencing-based approaches for accurate Brucella strain typing. WGS enables high-resolution genotyping of bacterial isolates at the deepest taxonomic level as well as characterization of bacterial pathogens (69). WGS has been successfully used in outbreaks to illustrate transmission routes and pathogen spread and to assess the epidemiological linkage between isolates (69). Nevertheless, careful harmonization of sequence data generation and interpretation is essential to improve reproducibility and transferability of results across different sectors and disciplines. The cgMLST is a WGS genotyping system based on a predefined set of core genes that, on the one hand, provides superior resolution compared to classical methods. On the other hand, cgMLST has made it possible to establish a harmonized approach for bacterial genotyping in different clinical laboratories (33). In Brucella, genomic approaches based on cgMLST analysis have been successfully established only for B. melitensis (35, 36), which is the most commonly isolated Brucella species from humans. However, other Brucella species such as B. abortus and B. suis are frequently responsible for infections in humans and are associated with substantial economic losses in livestock production (70). In this study, we established a unified cgMLST scheme to characterize all pathogenic Brucella species in humans and animals. This scheme has been systematically developed to capture the diversity of all classical Brucella species, B. melitensis, B. abortus, B. suis, B. canis, B. ovis, B. neotomae, *B. ceti*, and *B. pinnipedialis*, as well as the nonclassical species B. microti and *B. inopinata* and the recently described Brucella strains from amphibians (4). This was initiated by performing a primary phylogenetic analysis of all available RefSeq Brucella genomes. The phylogenetic analysis was concordant with prior studies (1, 71), indicating that the different Brucella species form a distinct phylogenetic lineage and meet the criteria for classification into a single genomic species (Fig. 1). Similar observations on close relatedness between all classical Brucella species averaging core genome nucleotide identities of >99% and identical 16S rRNA and *recA* gene sequences were reported in earlier studies (72, 73).

The quality of sequenced genomes is a major concern for WGS-based methods, notably cgMLST. In order to minimize the effect of accidental incorporation of erroneous genomes on the scheme definition, we applied stringent quality filters on all genomes. We additionally followed the definition of soft-core genomes to select core targets. Therefore, we selected the genes that were present in more than 98% of the 612 Brucella genomes in the RefSeq database. A similar approach was used, for example, in Burkholderia pseudomallei (63) and Campylobacter jejuni/Campylobacter coli schemes (74), where a 95% threshold was used to select core genes. This approach has the advantage of allowing screening of a large number of initial genomes, including draft incomplete genome sequences. As a result, the final Brucella-wide scheme comprised 1,764 targets, which was consistent with previous estimations of the core genome (75) but included significantly more loci than the previously described 164-gene-based Brucella scheme (34).

The reproducibility of allele calling was found to depend on the quality of the assembled genomes, for which the sequencing depth was considered relevant for obtaining high-quality genomes (76, 77). For paired-end Illumina data, our results show that the sequencing depth affects the accuracy and rate of allele calling of cgMLST and can also lead to cluster mismatches that affect next-generation sequencing (NGS) diagnosis. The required depth threshold may also vary depending on the assembler used. For Brucella cgMLST, we recommend a depth of coverage of 40 or more for raw paired-end Illumina data assembled by SKESA or SPAdes (along with prior quality assessment of raw data for strain contamination and an average Q30 of no less than 80%). It is worth noting that the required quality criteria and depth of sequencing may be different for other sequencing platforms, e.g., Pacific Biosciences and Oxford Nanopore Technologies (78). Additionally, we have shown that the reproducibility of cluster types across different assemblers is rather more possible than that of cgST profiles. Therefore, definition of cluster nomenclature when interpreting cgMLST data using predefined or hierarchical cluster thresholds should allow comparability of samples produced using distinct bioinformatics approaches while also revealing epidemiological links between isolates.

Genome-based analyses enable unambiguous taxonomic resolution for species identification and strain typing (69). Considering the long incubation period of brucellosis in humans and animals, the outbreak and source are often not identified until later confirmation of the diagnosis. Again, tracing with high-resolution genotypic methods is practically more valuable, especially in areas of endemicity where frequent exposure and repeated outbreaks, animal movements, and livestock trade are also common. In areas of endemicity where mixed agricultural practices are often adopted, infections with multiple Brucella species and genotypes may also be possible. Hence, the availability of a single scheme that covers multiple species of Brucella and has a resolution comparable to that of a single-species scheme is valuable to reduce the need to establish multiple separate cgMLST schemes for each species typed and also to improve accurate species diagnosis without the requirement of extensive phenotypic characterization.

The applicability of genomic analysis proved valuable for outbreak investigations in strains with documented and with missing epidemiological data (79), e.g., to identify the source of infection (80). In this study, we have also shown that the novel cgMLST had high discriminatory power and hence can resolve the closely related strains in outbreaks, making the new scheme suitable for epidemiological surveillance of pathogenic Brucella. The cgMLST was clearly superior to the classical MLST in terms of resolution. The results of cgMLST were comparable to those of core genome-based SNP typing in outbreaks. This was observed based on 37 B. melitensis and 76 B. abortus outbreak strains (45), leading to similar conclusions as in the original studies.

Whole-genome-based bacterial genotyping methods involving the inference of single point mutations (SNP typing) or the detection of gene variants in the form of numbered allele profiles (cgMLST allele typing) are considered compatible methods with different advantages. The SNP typing methods allow robust phylogenetic reconstruction of SNP alignment data as they are based on base-by-base comparison and consider evolutionary models with masked recombination sites and mobile elements. The allele typing methods, on the other hand, are more likely to produce concordant and comparable results between laboratories since the typing data are easily portable via online nomenclature databases, and the underlying workflow is less computationally intensive and does not require complex bioinformatics skills. There is also the possibility to maximize the resolution of allele typing methods by including the core and accessory genes (whole-genome MLST). However, the incorporation of accessory genes faces several hurdles related to the versatile presence of accessory genes and accuracy in distinguishing core from accessory genes and in resolving orthologous and paralogous genes (81).

In conclusion, we have developed a new cgMLST scheme for Brucella strains. The new scheme can be a valuable tool for the study of brucellosis outbreaks and should represent a valuable genomic resource, especially for countries with endemic brucellosis. In turn, better tracing of strains and their genotyping from animal and human hosts will contribute to a better surveillance system that will support the implementation of a better control program.
