# Supplementary material for: Core Genome Multilocus Sequence Typing Scheme for Improved Characterization and Epidemiological Surveillance of Pathogenic Brucella
Source: J Clin Microbiol. 2022 Jul 19;60(8):e00311-22. doi: 10.1128/jcm.00311-22 (PMC9387271; doi:10.1128/jcm.00311-22)
Supplement: Supplemental file 1 — Figs. S1 - S3. Download jcm.00311-22-s0001.pdf, PDF file, 2.1 MB [file jcm.00311-22-s0001.pdf]

Supplemental Material (Supplemental Figures S1 – S3)

Core genome multilocus sequence typing scheme for improved characterization and epidemiological surveillance of pathogenic *Brucella*

Mostafa Y. Abdel-Glil [a,b,c], Prasad Thomas [d], Christian Brandt[c], Falk Melzer[a], Anbazhagan Subbaiyan[d], Pallab Chaudhuri[d], Dag Harmsen[e], Keith A. Jolley[f], Anna Janowicz[g], Giuliano Garofolo[g], Heinrich Neubauer[a], Mathias W. Pletz[c]

[a]Institute of Bacterial Infections and Zoonoses, Friedrich-Loeffler-Institut, Naumburger Str. 96A, 07743 Jena, Germany.
[b]Faculty of Veterinary Medicine, Zagazig University, Sharkia province, Egypt.
[c]Institute for Infectious Diseases and Infection Control, Jena University Hospital – Friedrich Schiller University, Jena, Germany.
[d]Division of Bacteriology and Mycology, ICAR-Indian Veterinary Research Institute, Izatnagar, 243122, India.
[e]Department of Periodontology and Operative Dentistry, University Hospital Muenster, Muenster, Germany
[f]Department of Zoology, University of Oxford, Oxford, United Kingdom.
[g]Istituto Zooprofilattico Sperimentale dell'Abruzzo e del Molise "G. Caporale", Teramo, Italy

Supplemental Figures S1 – S3

Supplemental Figure S1: A maximum-likelihood (ML) phylogenetic tree computed with RAxML based on 159,572 SNP positions identified in the 612 *Brucella* genomes using the NASP pipeline. Branch coloration highlights bootstrap values as indicated in the legend

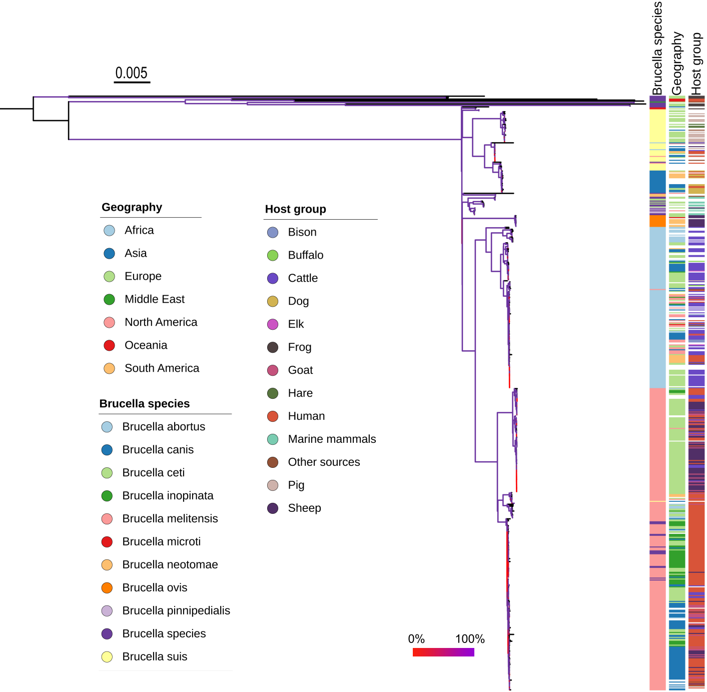

0.005
Brucella species
Geography
Host group
Geography
Africa
Asia
Europe
Middle East
North America
Oceania
South America
Host group
Bison
Buffalo
Cattle
Dog
Elk
Frog
Goat
Hare
Human
Marine mammals
Other sources
Pig
Sheep
Brucella species
Brucella abortus
Brucella canis
Brucella ceti
Brucella inopinata
Brucella melitensis
Brucella microti
Brucella neotomae
Brucella ovis
Brucella pinnipedialis
Brucella species
Brucella suis
0%
100%

Supplemental Figure S2: Minimum spanning tree (MST) calculated for 37 *B. melitensis* genomes with known epidemiological relation using the *B. melitensis*-specific core genome MLST targets (n= 2,704). The MST was generated using Ridom SeqSphere v07 with missing values being ignored in pairwise comparisons. Unique cgMLST profiles are represented with circles. The colors of the circles represent the genetic group of strains, while the circle size is related to the number of genomes assigned in each cgMLST genotype. The number of different alleles between cgMLST profiles is indicated on the connecting lines between the circles. The solid and dashed connecting lines represent allele differences below and above 10, respectively. Clusters were identified based on fewer than three allele mismatches and highlighted with grey shaded areas.

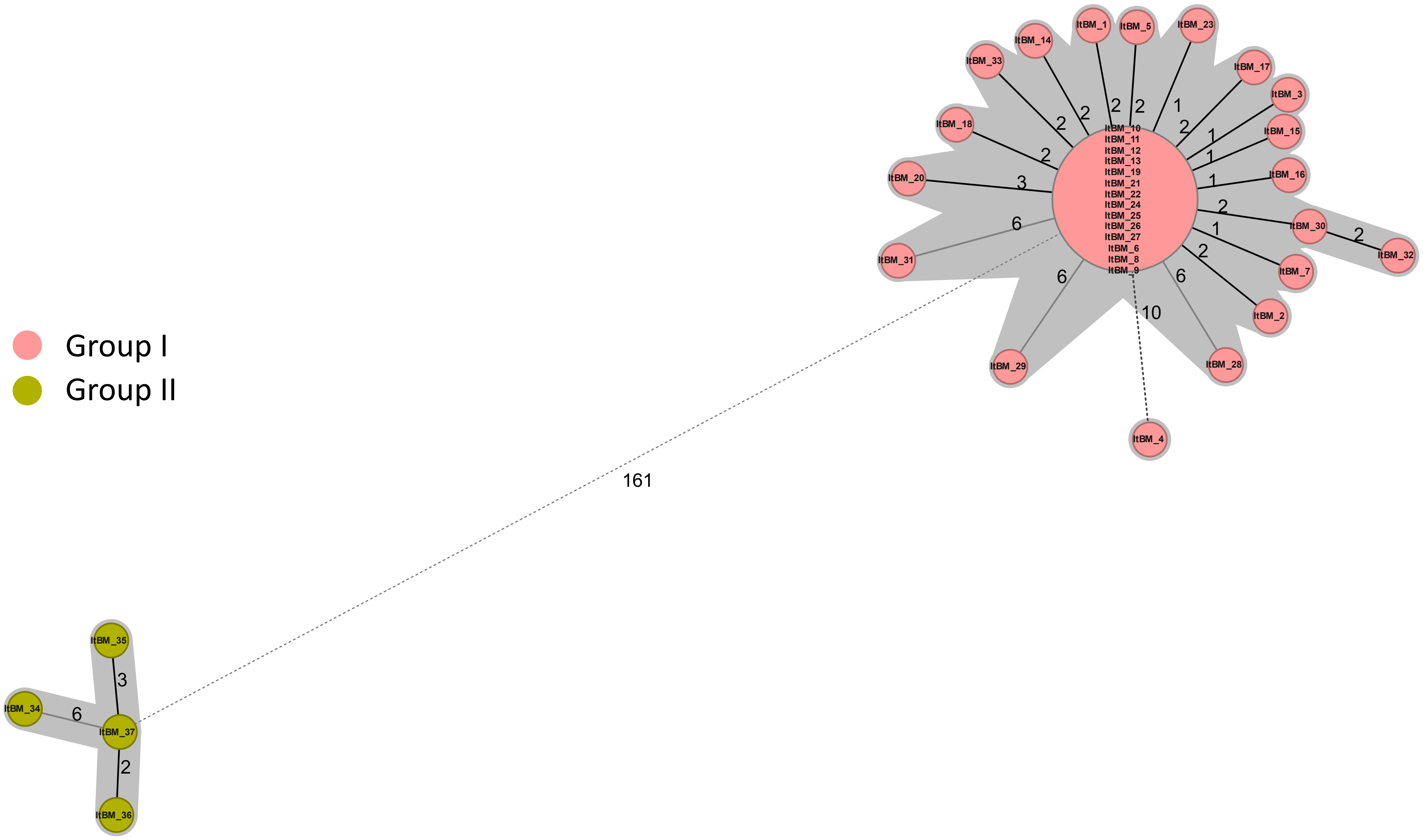
Group I
Group II
ItBM_10
ItBM_11
ItBM_12
ItBM_13
ItBM_19
ItBM_21
ItBM_22
ItBM_24
ItBM_25
ItBM_26
ItBM_27
ItBM_6
ItBM_8
ItBM_9
ItBM_1
ItBM_5
ItBM_23
ItBM_14
ItBM_33
ItBM_17
ItBM_3
ItBM_15
ItBM_16
ItBM_18
ItBM_20
ItBM_30
ItBM_32
ItBM_31
ItBM_7
ItBM_2
ItBM_29
ItBM_28
ItBM_4
161
ItBM_35
ItBM_34
ItBM_37
ItBM_36

Supplemental Figure S3: A maximum-likelihood (ML) phylogenetic tree computed with RAxML for the 1,322 *Brucella* genomes. Branch coloration highlights bootstrap values as in Fig S1.

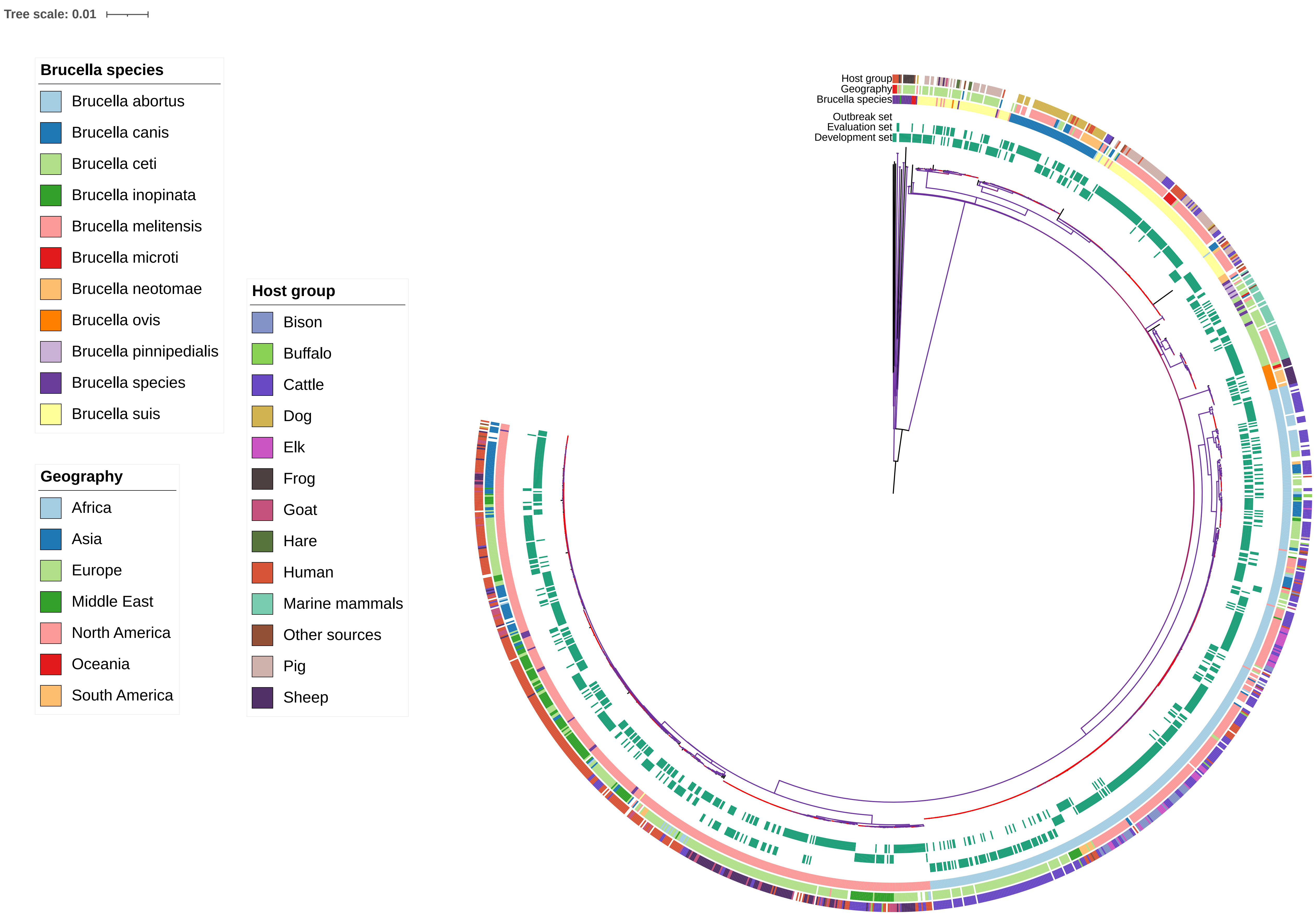
Tree scale: 0.01
Brucella species
Brucella abortus
Brucella canis
Brucella ceti
Brucella inopinata
Brucella melitensis
Brucella microti
Brucella neotomae
Brucella ovis
Brucella pinnipedialis
Brucella species
Brucella suis
Geography
Africa
Asia
Europe
Middle East
North America
Oceania
South America
Host group
Bison
Buffalo
Cattle
Dog
Elk
Frog
Goat
Hare
Human
Marine mammals
Other sources
Pig
Sheep
Host group
Geography
Brucella species
Outbreak set
Evaluation set
Development set
